# The PI3K/AKT/mTOR Pathway and Oral Diseases: A Bibliometric Analysis From 2008 to 2025

**DOI:** 10.1002/cre2.70238

**Published:** 2025-10-07

**Authors:** Yena Gan, Duoduo Li, He Xu, Sheng Han, He Zhu, Zening Wei, Zhigang Cai, Jinwei Huang

**Affiliations:** ^1^ Department of Academic Research International Research Center for Medicinal Administration Peking University Beijing People's Republic of China; ^2^ Department of Tuina and Pain Dongzhimen Hospital Beijing University of Chinese Medicine Beijing People's Republic of China; ^3^ National Center for Stomatology & National Clinical Research Center for Oral Diseases & National Engineering Research Center of Oral Biomaterials and Digital Medical Devices Beijing People's Republic of China; ^4^ Department of Pediatric Dentistry Peking University School and Hospital of Stomatology Beijing People's Republic of China; ^5^ Institute of Medical Innovation and Research & Medical Research Center Peking University Third Hospital Beijing People's Republic of China; ^6^ Department of Oral and Maxillofacial Surgery Peking University School and Hospital of Stomatology Beijing People's Republic of China; ^7^ Department of General Dentistry 2 Peking University School and Hospital of Stomatology Beijing People's Republic of China

**Keywords:** bibliometric analysis, pathogenesis, PI3K/AKT/mTOR, targeted therapy

## Abstract

**Objectives:**

The phosphoinositide 3 kinase (PI3K)/protein kinase B (AKT)/mammalian target of rapamycin (mTOR) signaling pathway is a key therapeutic target for oral diseases. This study uses bibliometric analysis to identify research trends, knowledge gaps, and the development of PI3K/AKT/mTOR‐targeted therapies.

**Methods:**

A systematic literature search was conducted in March 2025 using the keywords “PI3K/AKT/mTOR” and “dental OR oral.” Publication trends, highly cited studies, research hotspots, and emerging trends were analyzed. Comparative analyses of publication year, study design, and disease categories were performed between Asian and non‐Asian cohorts.

**Results:**

From 2008 to 2025, 119 studies were identified, with China, India, and Japan leading in publications and citations. Germany had the highest average citations. Keyword analysis showed a shift from basic to clinical research. Of these studies, 91 were from Asia, covering a broader range of oral conditions, while 28 were from non‐Asian regions, with no significant differences in study design or disease categories.

**Conclusion:**

This bibliometric analysis shows rising global interest in PI3K/AKT/mTOR research in oral science, led by Asia. Research has shifted from basic signaling to disease‐focused studies, with future efforts focusing on underexplored oral pathologies and translational applications, particularly in targeted therapy and regenerative medicine.

**Abbreviations**
AKT: AKT serine/threonine kinase
CCD: cleidocranial dysplasia
CDK4/6: cyclin‐dependent kinase 4/6
CDKN2A: cyclin‐dependent kinase inhibitor 2A
CMT1A: Charcot−Marie−Tooth disease type 1A
COL‐1: collagen type I
COX‐2: cyclooxygenase‐2
EGFR: epidermal growth factor
EMT: epithelial‒mesenchymal transition
FAK: focal adhesion kinase
FOXO: forkhead box protein O
GLUT1: glucose transporter 1
HBPC: hamster buccal pouch carcinoma
HIF‐1α: hypoxia‐inducible Factor 1α
HPV: human papillomavirus
IF: impact factor
IGF‐1: insulin‐like growth Factor 1
iNOS: inducible nitric oxide synthase
JCR: Journal Citation Reports
MDM2: mouse double minute 2
mIAS: mTOR inhibitor‐associated stomatitis
miR‐21: microRNA‐21
MRONJ: medication‐related osteonecrosis of the jaw
NF‐κB: nuclear factor kappa‐light‐chain‐enhancer of activated B cells
OSCC: oral squamous cell carcinoma
OSF: oral submucous fibrosis
PDL: periodontal ligament
PDLSC: human periodontal ligament stem cell
PIK3CA: phosphoinositide‐3‐kinase catalytic subunit α
PIP2: phosphatidylinositol‐bisphosphate
PIP3: phosphatidylinositol‐triphosphate
PTEN: phosphatase and tensin homolog
ROS: reactive oxygen species
RTKs: receptor tyrosine kinases
SRC: proto‐oncogene tyrosine‐protein kinase Src
TGF‐ß: transforming growth factor ß
TP53: tumor protein p53
VEGF: vascular endothelial growth factor
WoSCC: Web of Science Core Collection

## 1 Introduction

The phosphoinositide 3 kinase (PI3K)/protein kinase B (AKT)/mammalian target of rapamycin (mTOR) signaling pathway, which is activated by growth factors, including epidermal growth factor (EGF) and fibroblast growth factor (FGF), orchestrates a sequential cascade involving phosphatidylinositol‐triphosphate (PIP3), AKT, and mTOR, thereby regulating critical cellular processes (Hemmings and Restuccia 2012). This pathway governs gene expression, protein synthesis, cell proliferation, and cell cycle progression while simultaneously suppressing apoptosis and autophagy, thereby maintaining cellular homeostasis and facilitating disease progression (Rascio et al. 2021; González‐Moles et al. 2022). Given its hyperactivation in various malignancies and its pivotal role in disease metabolism and therapeutic resistance, the PI3K/AKT/mTOR pathway has emerged as a promising target for drug development and precision medicine treatment (Ketabat et al. 2019; Hoxhaj and Manning 2020; Cham et al. 2021). For example, the Rho‐associated protein kinase inhibitor Y‐27632 modulates the AKT/mTOR pathway and effectively induces autophagy, demonstrating its broader potential in treating oral pathologies involving dysregulated cell survival (Z. M. Wang et al. 2016). Co‐Fc‐coated nanoparticles have been shown to inhibit autophagy in oral squamous cell carcinoma (OSCC) cells, providing a targeted delivery system to suppress disease progression (Chen et al. 2023).

The PI3K/AKT/mTOR signaling pathway is involved in a wide spectrum of oral diseases, including OSCC, oropharyngeal squamous cell carcinoma (OPSCC) (Won et al. 2012), oral epithelial dysplasia (OED) (Martins et al. 2016), oral submucous fibrosis (OSF) (Adtani et al. 2024), periodontitis (Zhao et al. 2020), medication‐related osteonecrosis of the jaw (MRONJ) (Q. Wang et al. 2019), cleidocranial dysplasia (CCD) (Aonuma et al. 2020), Charcot−Marie−Tooth disease type 1 A (CMT1A) (Fledrich et al. 2014), and mTOR inhibitor‐associated stomatitis (mIAS) (Sonis et al. 2017). Genomic investigations have established that the PI3K/AKT/mTOR signaling pathway represents one of the most frequently altered oncogenic cascades in head and neck squamous cell carcinoma (HNSCC), with phosphoinositide‐3‐kinase catalytic subunit α (PIK3CA) mutations detected in approximately 30% of human papillomavirus (HPV)‐positive oropharyngeal tumors and phosphatase and tensin homolog (PTEN) loss observed in both precancerous and malignant lesions (Simpson et al. 2015). These findings suggest that dysregulation of this pathway occurs during the early stages of oral carcinogenesis, highlighting its significance beyond fully developed malignancies. While this pathway is implicated in diverse pathological processes, such as disease progression, inflammation, fibrosis, and tissue remodeling, the intricate network of interactions and disease‐specific regulatory mechanisms involved remains poorly understood. Despite extensive research, investigations into the PI3K/AKT/mTOR pathway in oral diseases are often fragmented, with disparate molecular targets, therapeutic strategies, and experimental models lacking comprehensive integration. This inherent complexity underscores the challenge of establishing unified research priorities and translational pathways. To address these issues systematically, bibliometric analysis has emerged as a valuable tool, enabling the mapping of publication trends, keyword co‐occurrence patterns, and emerging topic trajectories.

Bibliometric analysis is an objective and reliable method that employs statistical techniques to extract quantitative insights from extensive publications, offering a systematic, transparent, and reproducible overview of a scientific domain. It identifies research trends, knowledge structures, and clinical hotspots, facilitates intervention comparisons, and promotes collaborative efforts. It has been increasingly applied in dentistry, which provides valuable guidance for clinical practice and future research directions aimed at enhancing patient well‐being (Huang, Gan, Li, et al. 2025; Gan et al. 2025; Huang, Gan, Xu, et al. 2025; Huang et al. 2024; Aria and Cuccurullo 2017). The purpose of this study was to identify research hotspots, delineate knowledge gaps, and clarify the development trajectory of PI3K/AKT/mTOR‐targeted therapeutic strategies in oral diseases with the assistance of the bibliometric tools, thereby facilitating more focused and evidence‐driven future research endeavors.

## 2 Methods

A comprehensive literature search of titles and abstracts using the keywords “PI3K/AKT/mTOR” and “dental OR oral” was conducted across the Scopus, Web of Science Core Collection (WoSCC), PubMed, Embase, and Cochrane Library databases on March 6, 2025 (Supporting Information Material 1). The research protocol was prospectively registered in the International Platform of Registered Systematic Review and Meta‐analysis Protocols (No. INPLASY202560066, see Supporting Information Material 2). After removing duplicates, two independent investigators (J.H. and Y.G.) conducted the initial screening of publications for eligibility based on predefined inclusion criteria. The full‐text articles were subsequently reviewed when necessary, and any discrepancies were adjudicated by a third independent reviewer (H.X.) to ensure consensus.

This bibliometric analysis exclusively incorporated studies that specifically examined the role of the PI3K/AKT/mTOR signaling pathway in the pathogenesis of oral diseases. The exclusion criteria were studies focused on systemic disorders, pharmacological treatments unrelated to dental or oral pathologies, postsurgical or posttraumatic repair mechanisms, and investigations of alternative signaling pathways that do not involve the PI3K/AKT/mTOR axis. A comprehensive data cleaning procedure, including the systematic removal of extraneous keywords (e.g., articles), was implemented before data extraction to increase accuracy. A predesigned Microsoft Excel spreadsheet was utilized to compile article‐specific metadata, encompassing publication year, authors, country/region, affiliations, title, journal, Journal Citation Reports (JCR) division, impact factor (IF), study designs, keywords, references, and citation counts. Furthermore, detailed information on disease classifications, therapeutic interventions, and critical components associated with the PI3K/AKT/mTOR signaling pathway was extracted.

**Figure 1:**
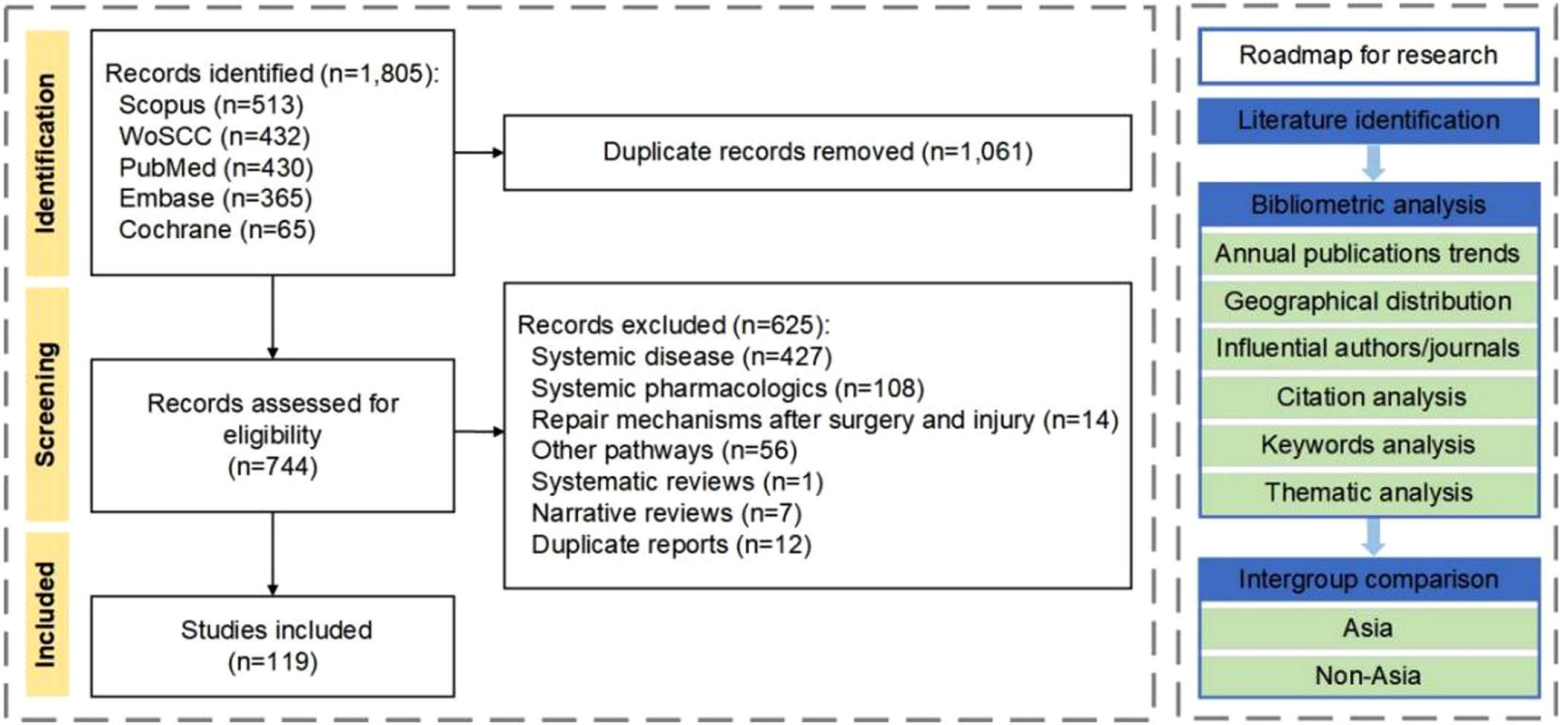
Flowchart of study selection and research methodology.

The annual and geographic distribution of publications was evaluated, alongside the identification of the most productive and influential journals, countries, and authors. Additionally, an in‐depth analysis was conducted to identify highly cited works, research hotspots, and emerging trends of study topics. Comparative analyses were performed to assess variations in publication years, study methodologies, and disease categories between Asian and non‐Asian cohorts. Bibliographic data were visualized and analyzed using the Bibliometrix and ggplot2 packages in R (version 4.3.1), complemented by VOSviewer (version 1.6.18) for network mapping. The study selection protocol and methodological framework are illustrated in a flowchart (Figure 1).

## 3 Results

**Figure 2:**
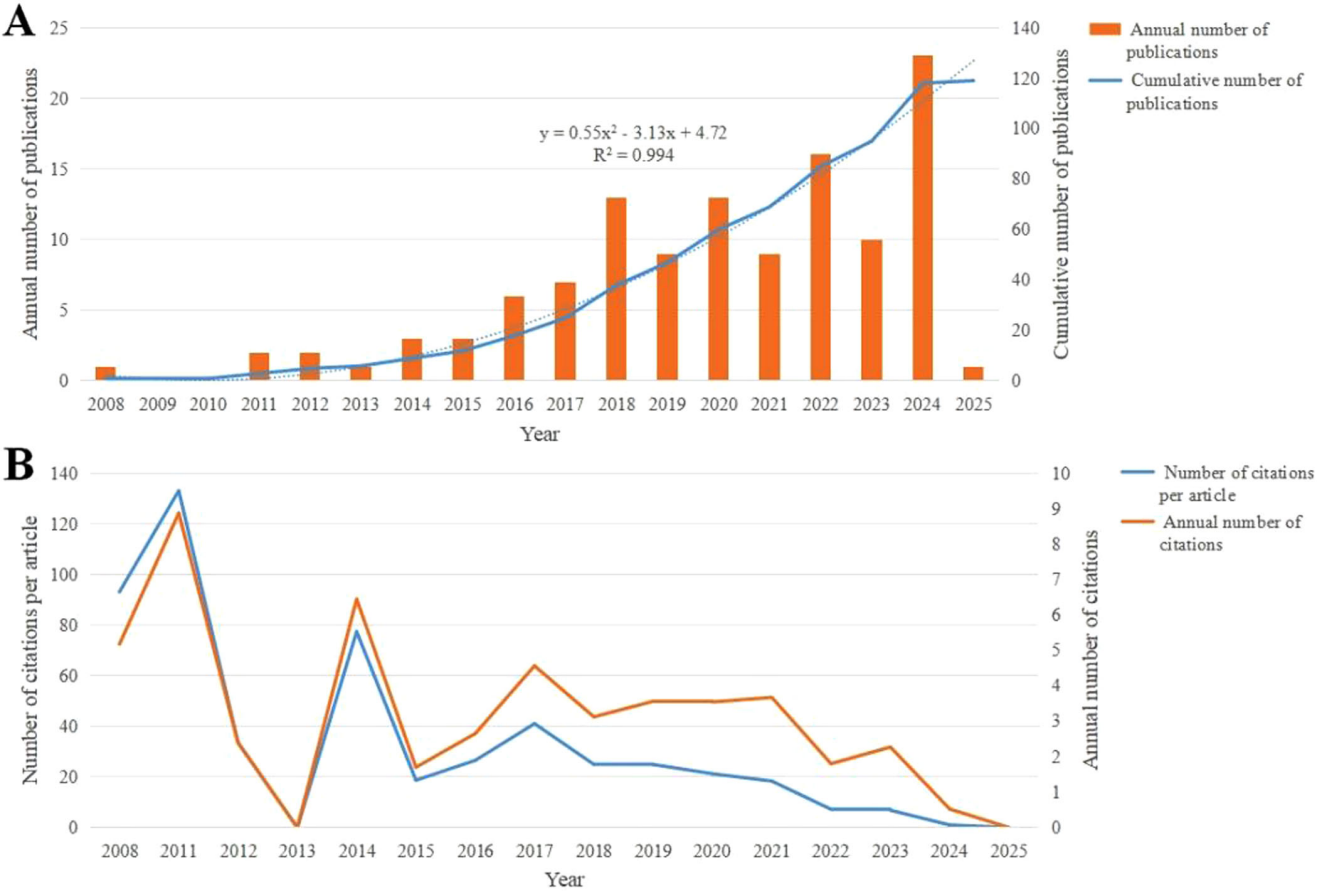
Trends in publication count and citation frequency. (A) Annual trends in publication count. (B) Annual trends in citation frequency.

After screening, 119 studies published between 2008 and 2025 met the inclusion criteria for bibliometric analysis (Figure 1). These publications were disseminated across 95 journals and authored by 685 individuals affiliated with 195 institutions spanning 13 different countries. The cumulative publication count demonstrated a nonlinear, accelerating growth pattern, best described by a quadratic polynomial model ($y=0.55x^{2}-3.13x+4.72$, $R^{2}=0.994$) (Figure 2A). Citation analysis revealed that both average annual citations and citations per article peaked in 2011, followed by a gradual decline (Figure 2B).

**Figure 3:**
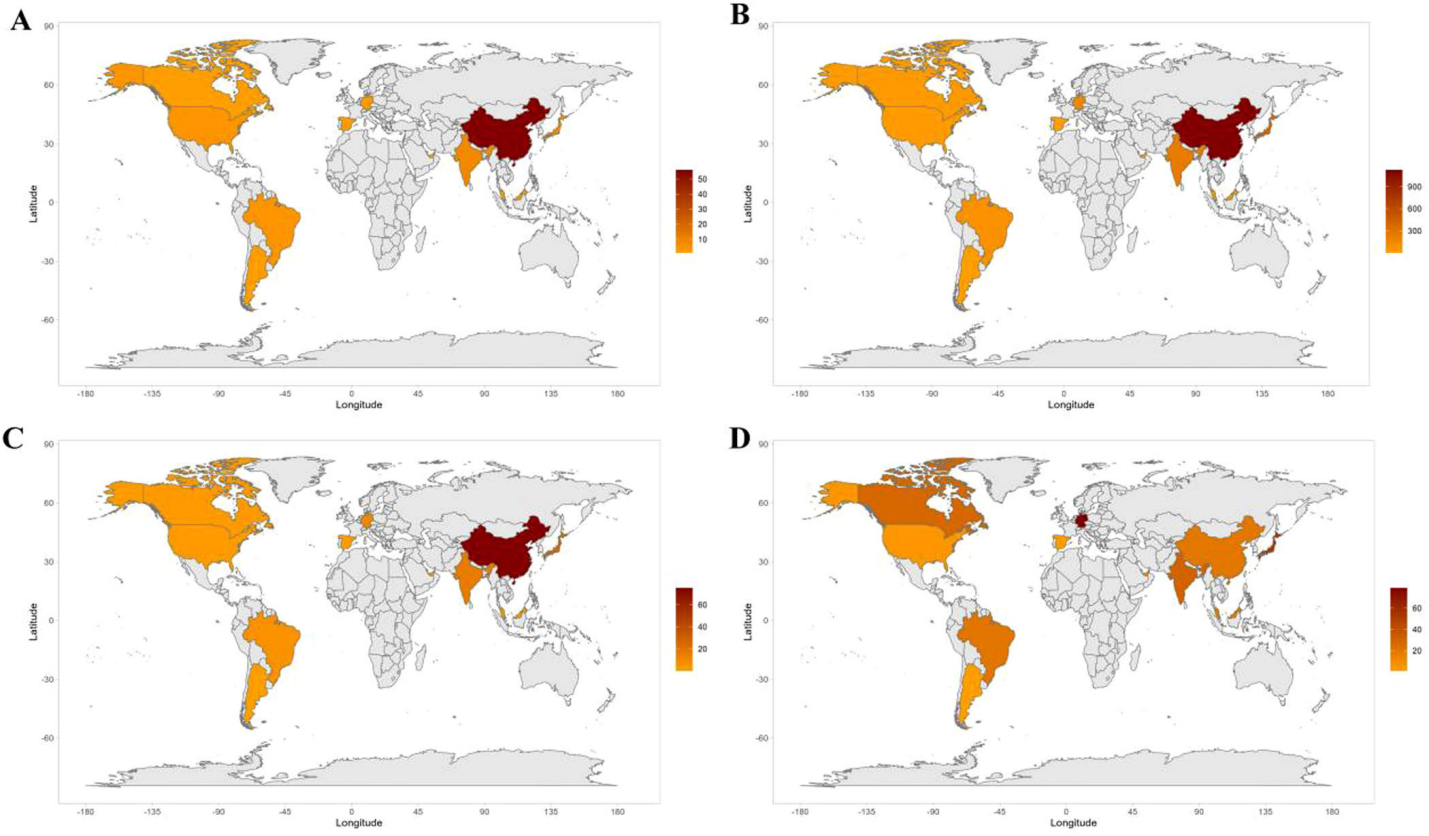
The geographic contributions of countries. (A) Overall publications. (B) Overall citations. (C) Mean citations per year. (D) Mean citations per publication.

Geographical distribution analysis identified China, India, and Japan as the leading contributors in terms of publication volume, total citations, and annual citations (Figure 3, Table 1). Notably, Germany presented the highest mean number of citations per article (mean = 79) (Figure 3). Institutional analysis revealed three prominent contributors: Shanghai Jiao Tong University School of Medicine (*n* = 33, China), China Medical University (*n* = 25, China), and Kyungpook National University (*n* = 22, Korea).

**Table 1:** Most productive and influential countries, authors, and journals.

Rank	Country/Region productivity (Publications)	Country/Region influence (Citations)	Author productivity (Publications)	Author influence (Citations)	Journal productivity (Publications)	Journal influence (Citations)
1	China (55)	China (1126)	Liu Y. (5)	Furuta M. (235)	*International Journal of Molecular Sciences* (7)	*Cancer Research* (235)
2	India (10)	Japan (388)	Liu H. (4)	Imoto I. (235)	*Journal of Cancer* (5)	*International Journal of Oncology* (228)
3	Japan (9)	India (243)	Park K.R. (4)	Inazawa J. (235)	*Biomedicine and Pharmacotherapy* (4)	*Nature Medicine* (148)
4	USA (9)	Germany (158)	Wang H. (4)	Kozaki K.I. (235)	*Oral Diseases* (4)	*Journal of Cancer* (101)
5	Taiwan (9)	Korea (98)	Wang L. (4)	Morita K.I. (235)	*Archives of Oral Biology* (2)	*Biomedicine and Pharmacotherapy* (94)
6	Korea (8)	Brazil (85)	Wang Y. (4)	Omura K. (235)	*Cancer Medicine* (2)	*BMC Cancer* (93)
7	Brazil (4)	Malaysia (58)	Yun H.M. (4)	Tsuruta T. (235)	*Drug Design, Development and Therapy* (2)	*Oncotarget* (86)
8	Malaysia (3)	Canada (28)	Cheong S.C. (3)	Uesugi A. (235)	*Heliyon* (2)	*Drug Design, Development and Therapy* (81)
9	Germany (2)	UK (18)	Kim E. (3)	Chang C.H. (173)	*International Journal of Oncology* (2)	*Cellular Physiology and Biochemistry* (66)
10	Spain (2)	USA (16)	Li C. (3)	Chiu H.Y. (173)	*Journal of Cellular Physiology* (2)	*Cell Death and Disease* (63)

The analysis indicated that 89.05% of the authors contributed to a single study. The most cited publication (*n* = 235) was authored by Uesugi et al. (2011) in *Cancer Research* (Q1, IF = 12.9, 2023) (Uesugi et al. 2011). The *International Journal of Molecular Sciences* (Q1, IF = 4.9, 2023) emerged as the most prolific journal, publishing seven included articles (Table 1).

The top 10 studies with ≥ 50 citations highlighted the pivotal role of the PI3K/AKT/mTOR signaling axis in the biology and therapeutic targeting of oral diseases (Table 2). Specifically, the modulation of this pathway through epigenetic, metabolic, pharmacological, and immunological strategies provided a multifaceted approach for improving OSCC outcomes. For example, epigenetic regulation, such as deoxyribonucleic acid hypermethylation, silenced tumor‐suppressive microRNAs (e.g., miR‐218 and miR‐585), which normally inhibited key oncogenic components of the mTOR complex (Uesugi et al. 2011). In addition to OSCC, similar mechanisms were observed in peripheral nervous system disorders, such as CMT1A, where dysregulated PI3K/AKT signaling impaired Schwann cell differentiation (Fledrich et al. 2014). Additionally, early neuregulin‐1 therapy targeting this pathway demonstrated neuroprotective potential (Fledrich et al. 2014).

**Table 2:** Top 10 studies with ≥ 50 citations.

Study	Title	Journal	Citations
Uesugi et al. (2011)	The tumor suppressive microRNA miR‐218 targets the mTOR component Rictor and inhibits AKT phosphorylation in oral cancer	*Cancer Research*	235
Chang et al. (2017)	Resveratrol‐induced autophagy and apoptosis in cisplatin‐resistant human oral cancer CAR cells: A key role of AMPK and Akt/mTOR signaling	*International Journal of Oncology*	173
Fledrich et al. (2014)	Soluble neuregulin‐1 modulates disease pathogenesis in rodent models of Charcot‐Marie‐Tooth disease 1A	*Nature Medicine*	148
Chakraborty et al. (2008)	Involvement of TSC genes and differential expression of other members of the mTOR signaling pathway in oral squamous cell carcinoma	*BMC Cancer*	93
Yu et al. (2017)	Targeting the PI3K/AKT/mTOR signaling pathway as an effective radiosensitizing strategy for treating human oral squamous cell carcinoma in vitro and in vivo	*Oncotarget*	68
Qi and Zhang (2014)	The microRNA 132 regulates fluid shear stress‐induced differentiation in periodontal ligament cells through the mTOR signaling pathway	*Cellular Physiology and Biochemistry*	66
Wei et al. (2018)	Salvianolic acid B inhibits glycolysis in oral squamous cell carcinoma via targeting PI3K/AKT/HIF‐1α signaling pathway	*Cell Death and Disease*	63
Harada et al. (2016)	Metformin in combination with 5‐fluorouracil suppresses tumor growth by inhibiting the Warburg effect in human oral squamous cell carcinoma	*International Journal of Oncology*	55
Li et al. (2019)	The immunoregulatory protein B7‐H3 promotes aerobic glycolysis in oral squamous carcinoma via the PI3K/Akt/mTOR pathway	*Journal of Cancer*	55
Aggarwal et al. (2019)	Targeted disruption of the PI3K/Akt/mTOR signaling pathway, via PI3K inhibitors, promotes growth‐inhibitory effects in oral cancer cells	*Cancer Chemotherapy and Pharmacology*	51

**Figure 4:**
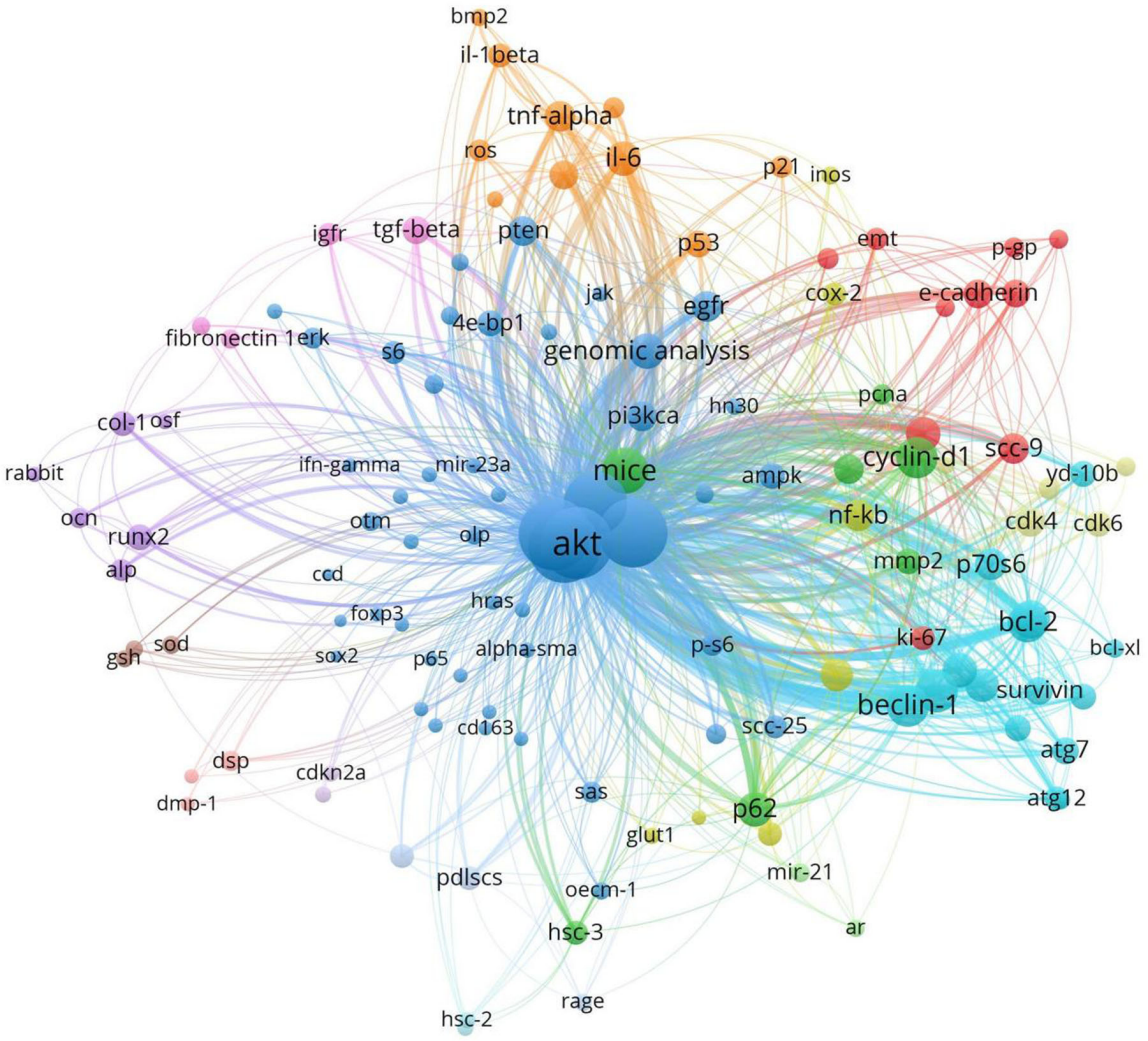
Keyword co‐occurrence map. The node size indicates the keyword frequency, and the line thickness represents the co‐occurrence frequency.

**Figure 5:**
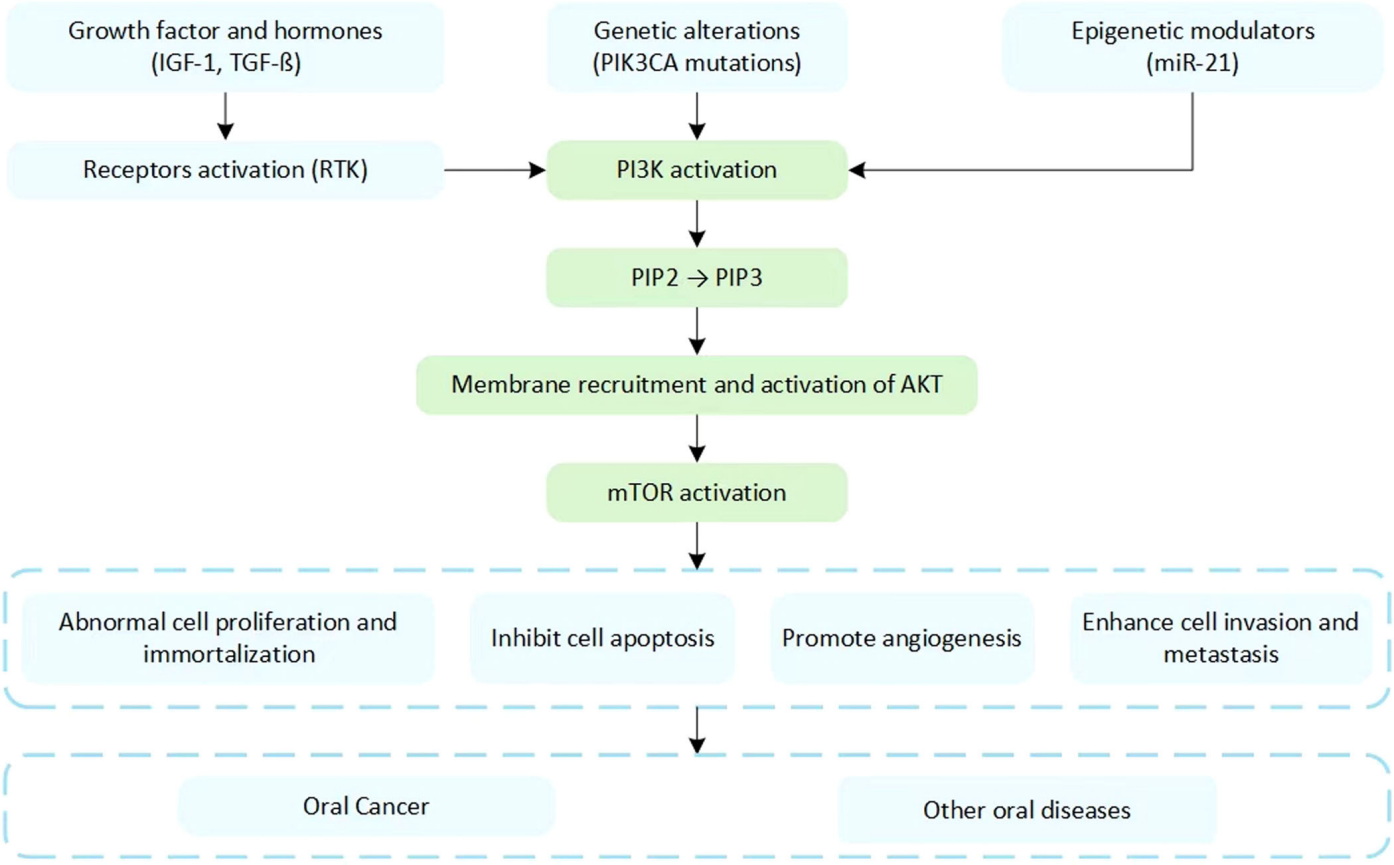
The overactivation of PI3K/AKT/mTOR in oral pathologies.

Keyword analysis revealed that the PI3K/AKT/mTOR signaling pathway is critically involved in diverse biological processes and pathological mechanisms related to oral diseases (Figure 4). The outcomes can be systematically categorized into four thematic domains. First, the PI3K/AKT/mTOR signaling pathway orchestrates key malignant behaviors in oral cancers, including cellular proliferation, epithelial‒mesenchymal transition (EMT), invasion, metabolic reprogramming, and chemoresistance, as evidenced in established cell lines such as CAL‐27, SCC‐9, HSC‐2, and HSC‐4 cells (Ji et al. 2021; Wei et al. 2020; Ito et al. 2016). Second, the pathway modulates tumor metabolism, inflammatory responses, and microenvironmental adaptation; it facilitates angiogenesis via vascular endothelial growth factor (VEGF)/hypoxia‐inducible factor 1‐α (HIF‐1α), promotes inflammation through nuclear factor kappa‐light‐chain‐enhancer of activated B cells (NF‐κB)/cyclooxygenase‐2 (COX‐2)/inducible nitric oxide synthase (iNOS), and regulates metabolic reprogramming via glucose transporter 1 (GLUT1) and reactive oxygen species (ROS) balance (Q. Wang et al. 2019; Aggarwal et al. 2019; Kishore T et al. 2016; Chang et al. 2017; Yu et al. 2017; Zainal et al. 2019; Hsu et al. 2018; Ganesan et al. 2024; Li et al. 2022; Qi and Zhang 2014; Wei et al. 2018; Shi et al. 2024). Third, the PI3K/AKT/mTOR axis regulates the balance between autophagy and apoptosis via molecular mediators such as LC3, Beclin‐1, Bcl‐2/Bax, caspases, and survivin, thereby modulating cell fate under therapeutic or stress conditions (Chang et al. 2017; Harada et al. 2016). Finally, the pathway involves extensive crosstalk with upstream and downstream modulators, including miR‐21, aldose reductase, cyclin‐dependent kinase 4/6 (CDK4/6), focal adhesion kinase (FAK), proto‐oncogene tyrosine‐protein kinase Src (SRC), tumor protein p53 (TP53), and cyclin‐dependent kinase inhibitor 2A (CDKN2A) (Uesugi et al. 2011; Yu et al. 2017; Zainal et al. 2019; Li et al. 2019). These factors collectively govern feedback loops, cell cycle control, and resistance mechanisms. Notably, these thematic clusters are interconnected; for example, EMT and apoptosis regulation converge in therapeutic studies; inflammatory and regenerative responses overlap in tissue repair processes; and molecular regulators form the backbone of signaling network integration. These findings highlight the PI3K/AKT/mTOR pathway as a highly integrated and multifunctional axis central to oral disease pathogenesis, immune modulation, stem cell differentiation, and therapeutic resistance (Figure 5).

**Figure 6:**
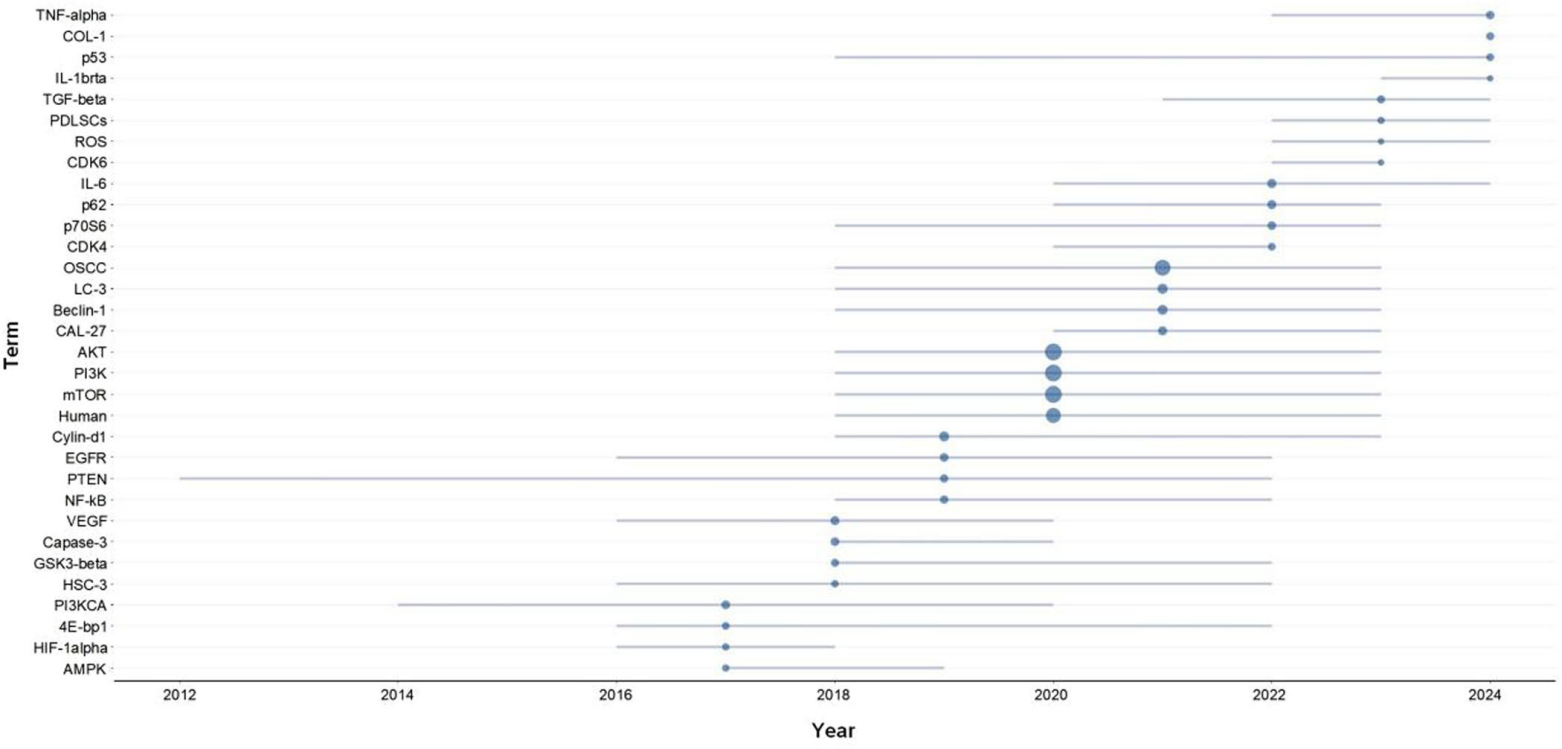
Trend topics. The horizontal lines and nodes represent the duration and median time of keyword appearance, respectively.

The temporal analysis of keyword trends revealed a distinct evolutionary pattern in PI3K/AKT/mTOR signaling pathway research on oral diseases (Figure 6). Initial investigations during the period of 2012–2016 predominantly focused on fundamental pathway components, including PI3K, AKT, mTOR, PTEN, and epidermal growth factor receptor (EGFR), indicative of a foundational emphasis on signal transduction mechanisms (Won et al. 2012). After 2018, the research scope broadened substantially, as evidenced by the increased frequency and density of keywords such as LC3, Beclin‐1, p62, p70S6K, IL‐6, CDK4/6, and caspase‐3, reflecting a transition toward exploring autophagy, apoptosis, inflammation, and cell cycle regulation (Ji et al. 2021; Wei et al. 2020; Aggarwal et al. 2019; Yu et al. 2017; Hsu et al. 2018). More recently (2022–2024), markers associated with bone formation (e.g., collagen type I [COL‐1] and human periodontal ligament stem cells [PDLSCs]), oxidative stress (e.g., ROS), and immune signaling (e.g., NF‐κB, interleukin 1ß, and tumor necrosis factor α) have gained prominence, suggesting an increasing focus on the involvement of the PI3K/AKT/mTOR signaling pathway in tissue regeneration, immune modulation, and the tumor microenvironment (Adtani et al. 2024; Ganesan et al. 2024; Li et al. 2022; Qi and Zhang 2014). Collectively, the analysis delineates a clear trajectory, that is, from molecular mechanistic studies to broader functional and therapeutic investigations, thereby positioning the PI3K–AKT–mTOR axis as a pivotal nexus in cancer biology, stem cell research, and inflammation‐related pathologies.

A comprehensive analysis of 119 studies revealed distinct geographical contributions, with 91 studies originating from Asian institutions and 28 from non‐Asian regions (Table 3). Comparative analysis revealed no statistically significant differences between these two groups in terms of publication year, study design, or disease type (*p* > 0.05). Both geographical cohorts exhibited a predominance of clinical investigations, followed by animal‐based experimental studies. OSCC emerged as the most extensively investigated pathological condition across both regions. The Asian research portfolio encompassed a diverse array of conditions, including CCD (Aonuma et al. 2020), hamster buccal pouch carcinomas (HBPCs) (Shi et al. 2024), MRONJ (Q. Wang et al. 2019), periodontitis (Zhao et al. 2020), periodontal ligament (PDL) disorders (Shi et al. 2024), hyperglycemia‐associated oral mucosal inflammation (Ganesan et al. 2024), and HPV‐induced oral inflammatory conditions (Lemmon and Schlessinger 2010). Conversely, non‐Asian studies focused primarily on CMT1A (Fledrich et al. 2014), mIAS (Sonis et al. 2017), and OSF (Adtani et al. 2024).

**Table 3:** Differences between the Asian and non‐Asian groups.

Characteristics	Asia	Non‐Asia	*p* value
Publication years	2020 ± 3.52	2019 ± 3.41	0.306
Study designs			0.564
Animal studies	29	6	
Clinical studies	47	17	
Genomic analyses	15	5	
Disease type			0.097
OSCC	75	21	
CCD	1	0	
CMT1A	0	1	
HBPC	1	0	
MRONJ	1	0	
mIAS	0	1	
Periodontitis	1	0	
PDL disorders	1	0	
Hyperglycemia‐associated oral mucosal inflammation	1	0	
OSF	0	1	
HPV‐induced oral inflammation conditions	1	0	
Multiple oral conditions	2	4	

Abbreviations: CCD, cleidocranial dysplasia; CMT1A, Charcot−Marie−Tooth disease type 1A; HBPC, hamster buccal pouch carcinomas; HPV, human papillomavirus; mIAS, mTOR‐inhibitor associated stomatitis; MRONJ, medication‐related osteonecrosis of the jaw; OSCC, oral squamous cell carcinoma; OSF, oral submucous fibrosis; PDL, periodontal ligament disorders.

## 4 Discussion

This study represents an inaugural bibliometric assessment of the PI3K/AKT/mTOR pathway in oral disease research, demonstrating a consistent upwards trajectory in publication volume accompanied by a gradual decline in citation rates since 2011. Three Asian countries, that is, China, India, and Japan, were identified as the most prolific contributors. Highly cited studies and keyword analysis elucidated the pivotal and expanding influence of pathways in oral pathology, particularly in disease progression, metabolic regulation, inflammatory processes, stem cell differentiation, and cellular apoptosis. The research trajectory has evolved from fundamental mechanistic studies of signaling components to broader functional implications and therapeutic advancements, demonstrating emerging relevance to additional conditions, including CMT1A (Fledrich et al. 2014). Asian researchers have contributed the predominant portion of scientific output, primarily focused on OSCC and a diverse spectrum of oral pathologies, whereas non‐Asian studies have explored a limited range of additional disorders. A comparison of regional contributions revealed no statistically significant differences in publication chronology, methodological approaches, or disease‐specific focus.

The PI3K/AKT/mTOR signaling pathway serves as a pivotal integrator that orchestrates extracellular signals with intracellular regulatory networks, significantly influencing fundamental biological processes, including stem cell differentiation. In dental‐derived stem cells, which include human dental pulp cells, dental pulp stem cells, and PDLSCs, the activation of the PI3K/AKT pathway through growth factors, including insulin‐like growth factor 1 (IGF‐1) and transforming growth factor ß (TGF‐ß), increases the expression of osteogenic and odontogenic markers (Backer 2010; Fedorova et al. 2022). In addition to its canonical activation via receptor tyrosine kinases (RTKs), such as EGFR and IGF receptors, the PI3K/AKT/mTOR signaling pathway is further modulated by genetic alterations (e.g., PIK3CA mutations) and epigenetic modulators such as miR‐21 (Rascio et al. 2021; Fedorova et al. 2022). Downstream, PI3K/AKT modulates key oncogenic mediators, including CDK4/6, which promote the G1‒S phase transition via cyclin D1 and p27 localization; it also modulates nonreceptor kinases such as SRC and FAK, which enhance cellular motility and invasion by integrating cytoskeletal remodeling to promote EMT (Fedorova et al. 2022). Furthermore, the AKT‐mediated phosphorylation of mouse double minute 2 (MDM2) inhibits TP53 function, suppressing apoptosis, whereas frequent alterations in CDKN2A synergize with PI3K/AKT hyperactivation to deregulate cell cycle checkpoints (Rascio et al. 2021).

The PI3K/AKT/mTOR signaling pathway plays a central role in regulating the malignant phenotypes and adaptive mechanisms of OSCC. This pathway is frequently activated in OSCC and orchestrates critical cellular processes by modulating downstream effectors such as mTOR, glycogen synthase kinase 3 ß (GSK3ß), and forkhead box protein O (FOXO) (Kim and Guan 2015). Functionally, it regulates cell fate by balancing autophagy and apoptosis, especially under therapeutic or stress conditions (Kim and Guan 2015). mTOR exerts inhibitory control over autophagy by suppressing autophagosome formation, while the downregulation of mTOR expression activates autophagic markers such as Beclin‐1 and LC3 (Alers et al. 2012). On the other hand, AKT suppresses apoptosis by inhibiting proapoptotic molecules such as Bad, Bax, and FOXO while upregulating the expression of antiapoptotic factors such as Bcl‐2, Bcl‐xL, and survivin (Dong et al. 2023). Oxidative stress can disrupt the Bcl‐2‐Beclin‐1 complex, triggering autophagy‐mediated or mitochondria‐linked apoptotic responses (Decuypere et al. 2012). Furthermore, this pathway facilitates EMT through the modulation of PIP3, β‐catenin, and E‐cadherin, thereby enhancing invasion and metastasis via epithelial‒mesenchymal crosstalk (Decuypere et al. 2012). These effects have been experimentally validated in OSCC cell lines, including CAL‐27, SCC‐9, SCC‐25, and SCC‐4 cells (Vo et al. 2023). Additionally, the PI3K/AKT/mTOR pathway contributes significantly to the tumor microenvironment by driving metabolic reprogramming, angiogenesis, and inflammation; it promotes glucose uptake and aerobic glycolysis through the upregulation of GLUT1, adenosine triphosphate citrate lyase, hexokinase, and 6‐biphosphatase 2, thereby providing metabolic flexibility for disease progression (Fedorova et al. 2022). Hypoxic conditions further amplify its angiogenic effects by stimulating HIF‐1α and VEGF expression (Hoxhaj and Manning 2020; Fedorova et al. 2022). Inflammatory signaling is reinforced via NF‐κB activation, increasing COX‐2 and iNOS levels and sustaining a tumor‐promoting inflammatory milieu (Fedorova et al. 2022). Moreover, this pathway regulates oxidative stress by modulating antioxidant markers such as superoxide dismutase, glutathione, and malondialdehyde, helping maintain redox homeostasis under elevated ROS levels (Decuypere et al. 2012). Collectively, these multifunctional roles establish the PI3K/AKT/mTOR axis as a critical integrator of oncogenic signaling and a promising target in OSCC therapy.

In addition to its role in OSCC, the PI3K/AKT/mTOR signaling pathway has been implicated in a variety of other oral diseases, including MRONJ, OSF, periodontitis, oral mucositis, chronic inflammatory conditions such as oral lichen planus (OLP), chronic graft‐versus‐host disease, and chronic oral candidiasis, as well as mIAS. These diseases are associated with varying degrees of pathway activation or inhibition across these conditions. For example, in MRONJ, bisphosphonates such as zoledronic acid inhibit the EGFR/Akt/PI3K/mTOR axis, resulting in impaired angiogenesis and decreased oral keratinocyte viability (Q. Wang et al. 2019). In OSF, arecoline induces fibrosis through the activation of the TGF‐ß1‐mediated PI3K/AKT/mTOR pathway, whereas sulforaphane counters this effect via NRF2 upregulation (Adtani et al. 2024). In periodontitis and orthodontic tooth movement, mechanical force and bioactive compounds such as rutin and enamel matrix derivatives enhance PDLSC proliferation and osteogenic differentiation through this signaling axis (Chakraborty et al. 2008). Chronic inflammation‐related diseases exhibit consistent PI3K activation mediated by cytokines such as interleukin (IL)−6 and regulatory microRNAs, suggesting a link between persistent inflammation and oncogenic transformation (Martins et al. 2016; Zhao et al. 2020). Under hyperglycaemic conditions, the PI3K/mTOR pathway drives oxidative stress and inflammatory responses in oral mucosal cells, which are mitigated by platycodin D (Ganesan et al. 2024). Additionally, mIAS is characterized by epithelial damage and cytokine release triggered by mTOR inhibition (Sonis et al. 2017). Despite the well‐documented centrality of the PI3K/AKT/mTOR pathway in these disorders, the current understanding remains fragmented, with most studies focusing on isolated mechanisms or in vitro models. A comprehensive exploration of its systemic involvement in disease pathogenesis, progression, and therapeutic response is urgently needed. Future research should aim to delineate the regulatory network of this pathway across diverse disease contexts, thereby advancing our understanding of its biological significance and informing the development of targeted therapeutic strategies.

The investigation of the PI3K/AKT/mTOR signaling pathway in oral diseases has progressively transitioned from mechanistic studies to more comprehensive and translational research. Early investigations focused on delineating the activation cascade from RTKs to AKT and mTOR, elucidating their roles in proliferation, apoptosis, and metabolic reprogramming through downstream targets such as GSK3ß, mTORC1, FOXO, and eukaryotic translation initiation factor 4e‐binding protein 1 (Fang et al. 2011). As the field has advanced, research has expanded to encompass upstream genomic and epigenetic regulators, including PIK3CA mutations, PTEN loss, and microRNA‐mediated modulation, and their influence on autophagy, apoptosis, and drug resistance (Won et al. 2012; Uesugi et al. 2011; Shi et al. 2024). Subsequent studies emphasized the interplay between PI3K/AKT signaling and cellular stress responses, immune modulation, angiogenesis, and inflammatory mediators such as NF‐κB and COX‐2 (Aggarwal et al. 2019; Kishore T et al. 2016). Additionally, the pathway is involved in stem cell behavior and differentiation, particularly under hypoxic and oxidative conditions, further underscoring its pivotal role in broader pathological contexts (Li et al. 2022). This evolution has also driven translational advancements, exemplified by the development of PI3K, AKT, and mTOR inhibitors and their synergistic integration with radiotherapy, immunotherapy, and autophagy‐inducing agents, marking a significant shift from basic research to clinical applications (Zainal et al. 2019).

The geographic distribution of PI3K/AKT/mTOR pathway research in oral diseases mirrors both epidemiological patterns and institutional research priorities. The disproportionately high burden of OSCC in South Asia and Southeast Asia, attributed to prevalent exposure to established risk factors, including tobacco, alcohol, and betel quid, has driven intensive academic efforts in these regions (Ranganathan and Kavitha 2025). This has resulted in a substantial body of research focused on OSCC and associated oral lesions, such as epithelial dysplasia (Martins et al. 2016).

Recent bibliometric analysis of the PI3K/AKT/mTOR literature has identified three predominant research themes: clinical trials, molecular mechanisms, and pathological processes. These trends emphasize key areas of focus, including biomarker‐driven therapeutic strategies, PTEN‐related investigations, and combination therapies (Deng et al. 2024). Translating these insights into the context of oral pathologies could facilitate the integration of fragmented studies and strengthen the connection between molecular discoveries and clinical applications. Consistent with these observations, clinical trials in HNSCC have evaluated PI3K inhibitors (e.g., buparlisib, BYL719), AKT inhibitors (e.g., MK‐2206), and mTOR inhibitors (e.g., everolimus, temsirolimus), with efficacy outcomes varying based on tumor genetic profiles (Uesugi et al. 2011; Mihai et al. 2024). Preclinical evidence further suggests that dual PI3K/mTOR inhibition may enhance radio‐sensitivity and overcome therapeutic resistance, although cross‐talk with parallel pathways such as EGFR and mitogen‐activated protein kinase (MAPK) remains a significant challenge. These findings underscore the necessity of biomarker‐guided, combination therapeutic strategies in targeting PI3K/AKT/mTOR signaling in oral diseases.

## 5 Strengths and Limitations

This bibliometric analysis, while providing a comprehensive overview of research on the PI3K/AKT/mTOR pathway in the oral field, is subject to several limitations. First, the restriction to English‐language publications may have excluded high‐quality studies in other languages. Second, while citation counts and publication volume serve as proxies for scientific impact, they do not fully capture methodological rigor or clinical significance. Systematic reviews, through rigorous selection and critical appraisal, can validate or refine these patterns, ensuring that the emerging research hotspots are both methodologically robust and clinically relevant to oral disease management. Hence, a systematic review might be needed to further clarify this topic. Third, the limited citation accumulation of recently published, high‐impact studies may constrain their visibility in this analysis.

## 6 Conclusion

This bibliometric investigation elucidates the evolving landscape of PI3K/AKT/mTOR signaling pathway research in oral science, revealing a marked surge in scientific engagement predominantly driven by Asian nations. China, India, and Japan generated many publications, whereas Germany emerged as a significant contributor to high‐impact studies. The research trajectory has transitioned from fundamental signaling mechanisms to comprehensive functional explorations, with a notable emphasis on OSCC, inflammation, metabolic regulation, and stem cell dynamics. The findings highlight the emerging research hotspots, including apoptosis, autophagy, and PTEN‐related mechanisms for oral diseases. Integrative efforts are warranted for advancing translational research, particularly in the development of biomarker‐driven therapies, combination strategies, and regenerative approaches for oral disease management.

## Author Contributions

**Yena Gan:** methodology, software, investigation, writing – original draft, visualization. **Jinwei Huang:** conceptualization, validation, investigation, data curation, writing – original draft, project administration. **Duoduo Li:** validation, data curation, writing – review and editing. **He Xu:** methodology, investigation, writing – review and editing. **Sheng Han:** validation, writing – review and editing. **He Zhu:** validation, data curation. **Zening Wei:** validation, data curation. **Zhigang Cai:** conceptualization, resources, writing – review and editing. All authors read and approved the final manuscript.

## Conflicts of Interest

The authors declare no conflicts of interest.

## Supporting information


**Table 1:** Searching strategies.

Supporting material 2.

## Data Availability

All data generated or analyzed during this study are included in this published article and the supporting file.
