# Supplementary material for: The PI3K/AKT/mTOR Pathway and Oral Diseases: A Bibliometric Analysis From 2008 to 2025
Source: Clin Exp Dent Res. 2025 Oct 7;11(5):e70238. doi: 10.1002/cre2.70238 (PMC12502616; doi:10.1002/cre2.70238)
Supplement: Supplementary file 1 — Table 1: Searching strategies. [file CRE2-11-e70238-s001.docx]

**Table 1** Searching strategies

| **Database** | **Retrieval formula** | **Results (2025-03-06)** |
| --- | --- | --- |
| Scopus | #1 TITLE-ABS-KEY("phosphatidylinositol 3-kinas*" OR "phosphatidylinositol 3 kinas*" OR "pi3k*" OR "pi-3k*" OR "kinases pi3*" OR "ptdins3-kinas*" OR "pi3-kinas*")  #2 TITLE-ABS-KEY("akt*")  #3 TITLE-ABS-KEY("rapamycin*" OR "mtor*" OR "tor*")  #4 TITLE-ABS-KEY = (dental OR tooth OR teeth OR oral)  #5 LANGUAGE = (English)  #6 DOCTYPE = (Article)  #7 #1 AND #2 AND #3 AND #4 AND #5 AND #6 | 513 |
| Web of Science Core Collection (WoSCC) | #1 TS=("phosphatidylinositol 3-kinas*" OR "phosphatidylinositol 3 kinas*" OR "pi3k*" OR "pi-3k*" OR "kinases pi3*" OR "ptdins3-kinas*" OR "pi3-kinas*")  #2 TS=("akt*")  #3 TS=("rapamycin*" OR "mtor*" OR "tor*")  #4 TS = (dental OR tooth OR teeth OR oral)  #5 LA = (English)  #6 DT = (Article)  #7 #1 AND #2 AND #3 AND #4 AND #5 AND #6 | 432 |
| PubMed | #1 "phosphatidylinositol 3-kinas*"[Title/Abstract] OR "phosphatidylinositol 3 kinas*"[Title/Abstract] OR "pi3k*"[Title/Abstract] OR "pi-3k*"[Title/Abstract] OR "kinases pi3*"[Title/Abstract] OR "ptdins3-kinas*"[Title/Abstract] OR "pi3 kinas*"[Title/Abstract]  #2 "akt*"[Title/Abstract]  #3 "rapamycin*"[Title/Abstract] OR "mtor*"[Title/Abstract] OR "tor*"[Title/Abstract]  #4 "dental"[Title/Abstract] OR "tooth"[Title/Abstract] OR "teeth"[Title/Abstract] OR "oral"[Title/Abstract]  #5 English[Language]  #6 #1 AND #2 AND #3 AND #4 AND #5 | 430 |
| The Cochrane Library | #1 ("phosphatidylinositol 3-kinas*" OR "phosphatidylinositol 3 kinas*" OR "pi3k*" OR "pi-3k*" OR "kinases pi3*" OR "ptdins3-kinas*" OR "pi3-kinas*"):ti,ab,kw  #2 ("akt*"):ti,ab,kw  #3 ("rapamycin*" or "mtor*" or "tor*"):ti,ab,kw  #4 ("dental" OR "tooth" OR "teeth" OR "oral"):ti,ab,kw  #5 #1 AND #2 AND #3 AND #4 | 65 |
| Embase | #1 'phosphatidylinositol 3-kinas*':ti,ab,kw OR 'phosphatidylinositol 3 kinas*':ti,ab,kw OR 'pi3k*':ti,ab,kw OR 'pi-3k*':ti,ab,kw OR 'kinases pi3*':ti,ab,kw OR 'ptdins3-kinas*':ti,ab,kw OR 'pi3-kinas*':ti,ab,kw  #2 'akt*':ti,ab,kw  #3 'rapamycin*':ti,ab,kw OR 'mtor*':ti,ab,kw OR 'tor*':ti,ab,kw  #4 dental:ti,ab,kw OR tooth:ti,ab,kw OR teeth:ti,ab,kw OR oral:ti,ab,kw  #5 [english]/lim  #6 [article]/lim  #7 #1 AND #2 AND #3 AND #4 AND #5 AND #6 | 365 |
