# Supplementary material for: The PI3K/AKT/mTOR Pathway and Oral Diseases: A Bibliometric Analysis From 2008 to 2025
Source: Clin Exp Dent Res. 2025 Oct 7;11(5):e70238. doi: 10.1002/cre2.70238 (PMC12502616; doi:10.1002/cre2.70238)
Supplement: Supplementary file 2 — Supporting material 2. [file CRE2-11-e70238-s002.pdf]

INPLASY

PI3K/AKT/mTOR pathway and oral diseases: a bibliometric analysis from 2008 to 2025

INPLASY202560066
doi: 10.37766/inplasy2025.6.0066
Received: 17 June 2025
Published: 17 June 2025

Gan, YN; Huang, JW; Li, DD; Xu, H; Han, S; Zhu, H; Wei, ZN; Cai, ZG.

Corresponding author:
Yena Gan

yena.gan@pkuircma.org.cn

Author Affiliation:
Peking University.

ADMINISTRATIVE INFORMATION

Support - Research Foundation of Peking University School and Hospital of Stomatology (PKUSS20240105).

Review Stage at time of this submission - Formal screening of search results against eligibility criteria.

Conflicts of interest - None declared.

INPLASY registration number: INPLASY202560066

Amendments - This protocol was registered with the International Platform of Registered Systematic Review and Meta-Analysis Protocols (INPLASY) on 17 June 2025 and was last updated on 17 June 2025.

INTRODUCTION

Review question / Objective To identify research hotspots, gaps, and trends in PI3K/AKT/mTOR-targeted strategies for oral diseases, guiding more focused, evidence-based studies.

Condition being studied The PI3K/AKT/mTOR signaling pathway, primarily activated by growth factors, orchestrates critical cellular functions including gene expression, protein synthesis, proliferation, and survival, thereby maintaining cellular homeostasis. Its frequent hyperactivation in malignancies and pivotal role in metabolic regulation and therapeutic resistance have positioned it as a central target in drug discovery and personalized treatment strategies. In the context of oral and dental diseases, numerous agents, such as Erufosine, Y-27632, LB1, and natural compounds including surfactin and antrodia salmonea, have been shown to modulate this pathway, inducing autophagy, apoptosis, or enhancing sensitivity to conventional therapies. These findings collectively highlight the therapeutic potential of targeting PI3K/AKT/mTOR signaling in oral pathologies. However, despite its broad involvement across conditions such as OSCC, OED, OSF, and MRONJ, the pathway's disease-specific regulatory mechanisms remain insufficiently characterized, largely due to the fragmented and heterogeneous nature of existing research.

METHODS

Search strategy The Scopus, Web of Science Core Collection (WoSCC), PubMed, Embase, and Cochrane Library databases will be systematically searched using the keywords "PI3K/AKT/mTOR" and "dental OR oral". Only English-language articles published from database inception to March 6, 2025, will be included.

Gan et al. INPLASY protocol 202560066. doi:10.37766/inplasy2025.6.0066 Downloaded from https://inplasy.com/inplasy-2025-6-0066/

Participant or population Studies will be included if they involve human participants, animal models, or cell lines that are used to investigate oral or dental diseases, and specifically examine the role of the PI3K/AKT/mTOR signaling pathway in disease pathogenesis. Eligible populations include those affected by conditions such as oral squamous cell carcinoma, periodontitis, oral epithelial dysplasia, and related disorders. Studies will be excluded if they focus on non-oral/dental diseases, do not assess the PI3K/AKT/mTOR pathway, or use populations (human, animal, or cellular) unrelated to oral pathology. Additionally, studies involving healthy subjects or normal tissues without disease relevance will not be considered.

Intervention Studies will be included if they involve interventions or exposures that directly modulate the PI3K/AKT/mTOR signaling pathway in the context of oral or dental diseases. This includes genetic manipulation (e.g., gene knockdown or overexpression), pharmacological inhibitors or activators targeting PI3K, AKT, or mTOR, as well as natural compounds or therapeutic agents known to affect this pathway, provided their effects are assessed in relation to disease pathogenesis. Studies will be excluded if the intervention or exposure does not involve the PI3K/AKT/mTOR pathway, or if the pathway is only mentioned without being directly assessed or modulated. Additionally, studies focusing on other signaling pathways without clear mechanistic linkage to PI3K/AKT/mTOR, or those investigating general treatment effects without specifying pathway involvement, will not be considered.

Comparator This review does not have any comparators or controls.

Study designs to be included In vitro studies, animal studies, clinical studies, interventional studies, translational research.

Eligibility criteria Inclusion criteria: English, all publication dates. Exclusion criteria: none.

Information sources Scopus, Web of Science Core Collection (WoSCC), PubMed, Embase, and Cochrane Library databases.

Main outcome(s) As a bibliometric analysis, this study will include measurable indicators relevant to bibliometric evaluation. These include publication year, authorship, country or region of origin, institutional affiliations, journal name, Journal Citation Reports (JCR) division, impact factor (IF), number of citations, study design, author keywords, and references. Articles must also contain content related to the PI3K/AKT/mTOR signaling pathway in the context of oral or dental diseases to ensure topical relevance.

Quality assessment / Risk of bias analysis As this is a bibliometric analysis, formal risk of bias or quality assessment of individual studies is not applicable.

Strategy of data synthesis Quantitative synthesis will be applied to outcome measures such as the annual number of publications, citation counts, impact factors, and author productivity, using descriptive statistics and visualizations (e.g., bar charts, trend lines, and geographical maps). Bibliometric tools will be used to identify highly cited articles, productive journals, authors, and institutions. Co-occurrence and clustering analyses will be conducted to identify research hotspots and emerging trends. Comparative analyses between Asian and non-Asian cohorts will be performed using quantitative methods (e.g., cross-tabulation and chi-square tests) to assess differences in publication years, study methodologies, and disease categories.

Subgroup analysis Not applicable.

Sensitivity analysis Not applicable.

Language restriction English.

Country(ies) involved China.

Keywords PI3K/AKT/mTOR, Bibliometric analysis, Oral squamous cell carcinoma, Pathogenesis.

Contributions of each author

Author 1 - Yena Gan.
Email: yena.gan@pkuircma.org.cn
Author 2 - Jinwei Huang.
Email: huangjinwei@bjmu.edu.cn
Author 3 - Duoduo Li.
Email: tarako@163.com
Author 4 - He Xu.
Email: kqxuhe2004@126.com
Author 5 - Sheng Han.
Email: hansheng@bjmu.edu.cn
Author 6 - He Zhu.
Email: kevinzhu@bjmu.edu.cn
Author 7 - Zening Wei.
Email: 916234168@qq.com
Author 8 - Zhigang Cai.
Email: c2013xs@163.com

Gan et al. INPLASY protocol 202560066. doi:10.37766/inplasy2025.6.0066 Downloaded from https://inplasy.com/inplasy-2025-6-0066/
